# Psycho-Emotional Aspects of Pregnant Women Diagnosed with a Rare Disease: A Systematic Review

**DOI:** 10.3390/nursrep14040245

**Published:** 2024-11-06

**Authors:** Celia Cortés-Martín, Juan Carlos Sánchez-García, Beatriz Piqueras-Sola, Jonathan Cortés-Martín, Andrés Reinoso-Cobo, Jose Manuel Martínez-Linares, Raquel Rodríguez-Blanque

**Affiliations:** 1 Research Group CTS1068, Andalusia Research Plan, Junta de Andalucía, 18014 Granada, Spain; celia92cortes@gmail.com (C.C.-M.); jsangar@ugr.es (J.C.S.-G.); bpiquerassola@gmail.com (B.P.-S.); rarobladoc@ugr.es (R.R.-B.); 2 Department of Nursing, Faculty of Health Sciences, University of Granada, 18071 Granada, Spain; jmmartinezl@ugr.es; 3 Virgen de las Nieves, University Hospital, 18014 Granada, Spain; 4 Department of Nursing and Podiatry, Faculty of Health Sciences, University of Malaga—Teatinos, Arquitecto Francisco Peñalosa 3, 29071 Malaga, Spain; andreicob@uma.es; 5 San Cecilio University Hospital, 18071 Granada, Spain

**Keywords:** psycho-emotional aspects, pregnant woman, rare disease, mental health, childbirth

## Abstract

**Background**: Psycho-emotional aspects as a cross-cutting theme have gained relevance and scientific interest in recent years. Pregnant women diagnosed with a rare disease constitute a vulnerable population, experiencing psycho-emotional challenges due to their specific circumstances. It is essential that this group is informed and receives the proper to manage the physical, emotional, and psychological challenges linked to their condition. **Objectives**: The aim of this review is to understand how the diagnosis of a rare disease affects the psycho-emotional aspects of a pregnant woman. **Methods**: The research question posed is how does the diagnosis of a rare disease affect the psycho-emotional aspects of a pregnant woman? This systematic review has been carried out following the PRISMA model and has been registered in PROSPERO with registration number CRD42024558523. A literature search was conducted in the databases of Scopus, PubMed, Cinahl, Scielo, and the Cochrane Library. Articles were selected on the basis of the following inclusion criteria: publication in the last twenty years and all languages. **Results**: In the end, 28 articles were selected. The main results highlight that there is a negative impact on the psycho-emotional level in these patients, altering aspects such as anxiety, stress, social rejection, and self-stigma. **Conclusion**: The role of nursing in addressing this psycho-emotional dimension as a mediator between families and other branches of the health sciences environment stands out.

## 1. Introduction

The study of psycho-emotional aspects as a cross-disciplinary subject has gained scientific relevance and interest in recent years. This concept encompasses the innate components of human beings that involve both their psychology and emotions, including a wide variety of mental processes and emotional states that impact behavior, interpersonal relationships, and general well-being. It includes aspects such as self-esteem, motivation, perception of the world, social skills, stress management, and emotional resilience, among others [1].

The management and understanding of psycho-emotional aspects are crucial in disciplines such as nursing, psychology, psychotherapy, pedagogy, psychiatry, and human development, as they allow for greater comprehension of the cognitive, affective, and behavioral processes of individuals in various circumstances and environments [2].

The intersection of different fields of study, such as rare diseases, pregnant women, and psycho-emotional aspects, creates an interesting specific scenario that has not yet produced a substantial amount of scientific literature despite the recent growth in interest in it [3].

Focusing on the study field of pregnant women diagnosed with one of these diseases, a series of complications related to their circumstances become evident [4].

Rare diseases are characterized by their low prevalence in the population, affecting fewer than 1 in 2000 live births [5]. Currently, there are between 7000 and 8000 different pathologies, also known as minority or rare diseases. It is estimated that approximately 80% of these conditions have a genetic origin. The etiology of the remaining 20% has a metabolic component [6].

To comprehensively understand the management of rare diseases, it is essential to analyze in detail the various associated clinical aspects, as well as the particular circumstances that each disease entails and its implications for the patient’s family, the environment, and the patient themselves, along with the interactions between these elements. The main challenges faced by patients diagnosed with a rare disease include its chronic nature, degenerative character, and significant disabling potential. Therefore, these patients require comprehensive and multidisciplinary interventions and care [7].

The delay in diagnosis [8], which can range from 4 to 10 years on average, hinders clinical management and may contribute to the development of mental disorders in patients. To date, scientific knowledge has been generated for approximately 800 of these diseases. Active patient participation in decision-making is crucial in these cases, with family and environment being essential elements in comprehensive treatment. It is important to highlight the fundamental role of patient associations in the field of rare diseases [9].

Women with rare diseases who are pregnant face a series of unique and complex challenges throughout the process of pregnancy, childbirth, and the postpartum period [4]. Firstly, a lack of information and the scarcity of specific studies on managing these conditions during pregnancy can leave women navigating uncharted territory. Additionally, many of these rare diseases are chronic and may require careful management with medications that could pose risks to the developing fetus [10].

Some rare diseases can directly affect the pregnancy process [11]. These include Asherman’s syndrome [12], which causes uterine adhesions and increases the risk of miscarriage and complications during childbirth; Marfan syndrome [7], associated with cardiovascular problems that raise the risk of aortic dissection and premature birth; congenital hypoparathyroidism, which can cause low calcium levels and increase the risk of pre-eclampsia; antiphospholipid syndrome [13], which increases blood clotting and is linked to recurrent miscarriages; and hyperemesis gravidarum, which, although not rare, can cause dehydration and electrolyte imbalances. These conditions require specialized medical care during pregnancy to minimize risks to both the mother and the fetus [14].

In addition to medical and physical challenges, pregnant women facing a rare disease also have to deal with a range of psycho-emotional aspects, the central focus of this study [1]. Fear and anxiety about the impact of their condition on fetal development, as well as concerns about their own health and well-being during pregnancy and childbirth, are common. These concerns can be exacerbated by a lack of social support and limited understanding of their condition by friends, family, and healthcare professionals [15].

It is essential that pregnant women facing a rare disease receive adequate support to help them cope with the physical, emotional, and psychological challenges associated with their condition. Additionally, it is important that healthcare professionals attending to these women are adequately trained and aware of the unique needs they face [16]. This may involve greater education about rare diseases and their implications during pregnancy, as well as open and understanding communication with the patients. Furthermore, it is crucial that healthcare providers remain aware of the possibility of patients seeking professional help in the area of psycho-emotional functioning from a psychologist, psychotherapist, and/or psychiatrist, ensuring a comprehensive approach to their care.

It is worth highlighting the irreplaceable role of nursing in addressing the psycho-emotional aspects of pregnant women diagnosed with a rare disease. Through empathetic, educational, and coordinated care, nurses can significantly improve the quality of life and emotional well-being of these women, providing emotional support and fostering open communication. They provide personalized education about the disease and pregnancy, coordinate care between different health professionals, and offer ongoing support. In addition, they facilitate access to psychoeducational interventions, such as stress management techniques, which contribute to improving patients’ quality of life in a holistic way. Expanding research in this field is crucial for further improving care and outcomes for this vulnerable population [2].

The justification for this study becomes evident when understanding the context in which it develops. The world of rare diseases requires addressing a series of needs, including a better understanding of their environment. The widespread lack of knowledge on this specific subject necessitates a better understanding that will aid in managing these cases. Studying the psycho-emotional aspects of pregnant women diagnosed with a rare disease will have positive repercussions in different fields. On the one hand, it will benefit the scientific community, as the contribution of this knowledge will illuminate an area of study where there is not much scientific production. At the same time, understanding the psycho-emotional aspects of the study population will lead to better management and handling of the clinical situation by healthcare professionals involved in their care. Lastly, and most importantly, the impact of this study on the patients, their families, and their environment will be positive, helping them to better understand themselves, manage their emotions, and resolve conflicts that arise, thereby improving their emotional and physical quality of life.

The aim of this review is to understand how the diagnosis of a rare disease affects the psycho-emotional aspects of a pregnant woman. The research question posed is how does the diagnosis of a rare disease affect the psycho-emotional aspects of a pregnant woman?

## 2. Methodology

### 2.1. Review Protocol

The methodology used for the preparation of this report was a systematic review of the scientific literature published on the psycho-emotional aspects of pregnant women diagnosed with a rare disease. This review followed the Preferred Reporting Items for Systematic Reviews and Meta-Analyses (PRISMA) protocol, which consists of a 27-item checklist covering the most representative sections of an original article, as well as the process for developing these guidelines. This systematic review was conducted following a protocol available on the website: [http://www.crd.york.ac.uk/PROSPERO/] (accessed on 4 November 2024) with registration number CRD42024558523.

### 2.2. Eligibility Criteria

Articles published in the last twenty years that provided information on the psycho-emotional aspects of pregnant women diagnosed with a rare disease were selected, with no restrictions on the language of publication. Therefore, the inclusion criteria were articles published in the last 20 years and in any language. There were no language restrictions so as not to exclude articles on this very specific topic; all articles were reviewed and translated by experts in the specific language. In addition, all types of articles, systematic reviews, case descriptions, observational studies, qualitative studies, and clinical trials were considered. Given the scarcity of publications on the subject, a broad search was carried out so as not to discard information from the outset.

Reviews and primary studies were treated separately and with caution. Studies included in reviews were not considered as independent primary studies to avoid the duplication of data. Thus, systematic reviews were included to provide a comprehensive and generic analysis of the existing literature, and primary studies were only included if they were not part of the selected systematic reviews in order to have a specific perspective on the study topic and to avoid the double counting of evidence.

### 2.3. Sources of Information

The literature search was conducted in the databases of Scopus, PubMed, Cinahl, Scielo, and the Cochrane Library. The structured language used was derived from MeSH terms and Health Sciences Descriptors (DeCS). The descriptors used were emotional, pregnant women, and rare disease, with the Boolean operators AND and OR.

The choice of “rare disease” as a search term is based on the generality proposed in the objective, as this was to understand how the diagnosis of a rare disease affects the psycho-emotional aspects of a pregnant woman. We considered the diagnosis of rare diseases in general, not of any specific disease. Therefore, a generic term was used to include all rare diseases and not disease-specific terms that would bias the information in the manuscript.

### 2.4. Search Strategy

The following table (Table 1) shows the search strategy used to conduct this work, along with the date on which the search was performed.

### 2.5. Data Extraction Process

After conducting the search strategy, the articles found were transferred to the Mendeley web application using the Mendeley web importer tool. They were then organized into folders according to the database from which they were obtained, and all duplicates were removed.

Two reviewers independently examined the title, abstract, and keywords of each study identified in the search and applied the inclusion and exclusion criteria. For potentially eligible studies, the same procedure was applied to the full-text articles. Differences between the reviewers were resolved by a third reviewer.

### 2.6. Data Collection Process and Data Collected

After the initial search of the aforementioned databases, 724 articles were located. After applying the inclusion and exclusion criteria, the number was reduced to 292 articles. Subsequently, a total of 217 articles were excluded, after reading the title and abstract, leaving 75 works. Finally, after a complete reading and elimination of duplicates, 28 results were obtained for this review.

We searched for all results consistent with each outcome domain in each study for all measures, time points, and approaches. This broad search was carried out so as not to discard any results a priori due to the scarcity of publications related to the topic of study.

The Section 3 explains the article selection process in more detail.

### 2.7. Risk of Bias in Individual Studies

To conduct a methodological evaluation of the articles selected for this study, the design, methodology, and type of study of each work were analyzed to select the most specific methodological evaluation scale for each case.

For the reviews, the Amstar-2 (A Measurement Tool to Assess Systematic Reviews) methodological evaluation scale was used. Amstar-2 provides a comprehensive quality assessment, incorporating flaws that may have arisen due to incorrect review conduct. Amstar-2 includes 16 domains, presenting simple response options: “yes” when the standard was met; “no” if the standard was not met or the existing information was insufficient to respond; and “partial yes” in situations where partial adherence to the standard occurred. Although it does not provide an overall score, four levels of confidence emerge: high, moderate, low, and critically low.

The Newcastle–Ottawa Scale (NOS) is a tool used to assess the methodological quality of observational studies, specifically cohort and case–control studies. The NOS uses a “star” system to judge studies in three broad categories: selection, comparability, and outcomes (or exposure, in the case of case–control studies). Each of these categories include several specific items. This tool has a total of 8 items.

For qualitative studies, the CASP (Critical Appraisal Skills Programme) was used. CASP provides tools to assess the quality of different types of studies, including qualitative studies. CASP tools are designed to help researchers and healthcare professionals evaluate the validity, importance, and applicability of evidence in research.

Cochrane RoB 2 (Risk of Bias 2) is a tool designed to assess the risk of bias in the results of randomized clinical trials. The Cochrane RoB 2 tool is used to assess five main domains in randomized clinical trials; each domain includes a series of questions (called “signals”) that guide the assessment of risk of bias. These questions are answered with “yes”, “probably yes”, “probably no”, “no”, or “no information” and are used to make a judgment about whether the risk of bias in that domain is low, high, or whether there is any concern about the risk of bias. In total, there are 23 items or signals to consider depending on the context and the specific study design.

For case descriptions, the SCED (Rating Scale for Single-Participant Designs) was used. SCED consists of 11 items, 10 of which are used to evaluate methodological quality, and 1 is used for statistical analysis.

Using the different scales to evaluate the methodology of the articles selected for this review, the results obtained are presented in the following table (Table 2).

## 3. Results

**Figure 1:**
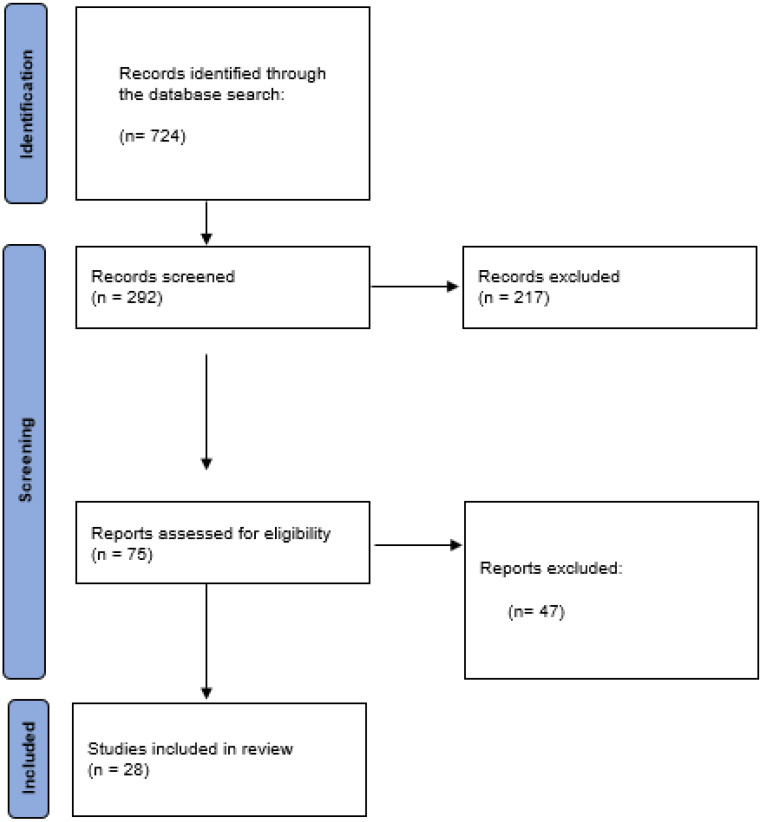
Flow diagram.

The flow chart resulting from the selection of articles for this systematic review is presented below (Figure 1).

The following table presents the main results obtained in this report (Table 3).

The psycho-emotional impact on pregnant women diagnosed with a rare disease is a complex topic that encompasses various dimensions. This review aims to explore the different studies that analyze this impact from various perspectives, with the goal of providing an integrated and deep understanding, considering the scarcity of published works on the subject.

Al-Hasan et al. [11] document a case of Susac syndrome in a pregnant woman, highlighting the diagnostic and therapeutic challenges this condition presents during pregnancy. The clinical management of Susac syndrome, a rare disease characterized by the triad of encephalopathy, retinopathy, and hearing loss, presents significant challenges due to the need to balance immunosuppressive therapy with risks to the fetus. Additionally, the emotional impact and how to approach motherhood with the presented limitations are emphasized.

The TRIDENT-2 study, conducted by Bakkeren et al. [17], evaluates the psychological impact of additional findings detected through non-invasive prenatal testing (NIPT). The results reveal that the detection of additional findings can cause considerable anxiety and stress in pregnant women, underscoring the need for proper genetic counseling and psychological support during this process.

On the other hand, Beauquier et al. [18] investigate the impact of twin-to-twin transfusion syndrome and monochorionicity on prenatal attachment and the mental health of mothers. These factors are found to be associated with higher levels of post-traumatic stress, anxiety, and depressive symptoms, highlighting the psycho-emotional vulnerability of these women.

A study by Betegón et al. [19] evaluates a program aimed at reducing anxiety in pregnant women diagnosed with small-for-gestational-age fetuses. The results show a significant decrease in anxiety levels following the intervention, highlighting the effectiveness of psychological support programs during pregnancy in the context of rare diseases.

Blok et al. [20] analyze the psychological impact of gestational trophoblastic disease in a multicenter cohort. The results indicate that this condition has a profound emotional impact, generating high levels of anxiety and depression, underscoring the importance of continuous psychological follow-up for these patients.

In the context of peripartum cardiomyopathy, Wolff et al. [21] conduct a qualitative study on the psychological adaptation of affected women. It is found that patients experience notable stress and emotional difficulties, requiring personalized interventions to support their mental and physical well-being.

Di Mattei et al. [22] address the psychological impact and defense mechanisms in women with gestational trophoblastic disease, highlighting the importance of understanding and supporting the coping strategies used by these patients during the course of the disease and follow-up. Dommergues et al. [23] explore the experiences of women with rare motor disabilities during childbirth and motherhood, finding that these women face unique challenges that affect their emotional well-being and require a specialized and sensitive care approach.

Studies on reproductive decisions, such as those by Dommering et al. [24] and Gregersen et al. [25], investigate decision-making in couples at risk of having children with retinoblastoma. The results show that the fear of transmitting the disease significantly influences the reproductive decisions and emotional well-being of the affected couples.

Hughes et al. [4] identify the psychosocial challenges of women with rare genetic conditions during pregnancy, revealing that these women often require additional support due to the uncertainty and stress associated with their condition.

The systematic review by Ireson et al. [26] on health-related quality of life in gestational trophoblastic disease highlights the need for patient-reported outcome measures to effectively address their psychological and emotional needs.

Johnson et al. [27] study the impact of pregnancy on women with myotonic dystrophy, finding that the condition exacerbates physical and emotional challenges, requiring a multidisciplinary care approach to adequately manage their needs. Qualitative studies on parent experiences when dysmelia is identified during the prenatal and perinatal periods, such as those by Johnson et al. [28,29], emphasize the importance of family-centered care and psychological support to address the feelings of guilt and anxiety of the parents.

Lekarev et al. [30] address the complications associated with adrenal diseases during pregnancy, highlighting the importance of careful management to minimize risks for both the mother and the fetus.

Marinello et al. [14] use a narrative medicine approach to explore the experiences and unmet needs of women with rare and complex connective tissue diseases during pregnancy, finding that these women often feel that their concerns are not adequately addressed by healthcare professionals.

Sherrell [31] highlights the importance of counseling for parents of children with rare diseases, emphasizing that anxiety and stress are prevalent and that psychological support can significantly improve parental adaptation and emotional well-being.

Smeltzer et al. [32] and Somanadhan et al. [33] investigate the perinatal experiences of women with physical disabilities and parents of children with mucopolysaccharidosis, respectively, highlighting the emotional transitions and challenges they face, as well as the importance of sensitive and understanding clinical care.

The case of Natasha, presented by Wilson et al. [34], illustrates the neuropsychological challenges of a woman with Sheehan’s syndrome and sickle cell disease during pregnancy, emphasizing the need for multidisciplinary support to manage the complex interactions between these conditions.

Finally, studies by Gómez López et al. [1] and Mills et al. [35] emphasize the common psychological alterations in pregnant women and the experiences of women with lymphoma during pregnancy, respectively, underscoring the prevalence of anxiety, stress, and the need for specific interventions to improve psycho-emotional well-being.

Special attention is given to highlighting the role of nursing in addressing these cases. Although not directly dedicated to an exclusive article, it is emphasized in several reviewed studies, such as [2,16,34]. These studies highlighted aspects such as the coordination between healthcare branches and the patient, emotional support, psychosocial support, and the development of personalized care plans to address the identified needs in each case.

## 4. Discussion

The aim of this review was to answer the following research question: how does the diagnosis of a rare disease affect the psycho-emotional aspects of a pregnant woman? Based on the results obtained and presented above, it is evident that the psycho-emotional impact on pregnant women diagnosed with rare diseases is a complex and multifaceted issue. Through the review of selected studies for this analysis, it is possible to outline a comprehensive and comparative view of the emotional and psychological challenges these women face, as well as effective support strategies.

It is noteworthy that the focus of the articles found on this topic follows a specific approach, aiming to relate the emotional impact of this specific population with a particular pathology. Some studies suggest that a more general perspective in addressing this issue would be more productive, understanding the psycho-emotional realm as something common and affected by all rare diseases to varying degrees [40].

This section discusses various aspects of the psycho-emotional impact on pregnant women diagnosed with rare diseases, focusing on three main themes from this systematic review: the anxiety generated by diagnosis and prenatal findings, the emotional impact of specific rare diseases during pregnancy, and the importance of psychological interventions and a multidisciplinary approach to improve their well-being. Below is a thematic organization summarizing the relevant findings in these areas.

One of the most emotionally impactful moments for pregnant women is receiving a diagnosis of a rare disease. The TRIDENT-2 study by Bakkeren et al. [17] explores the psychological impact of additional findings in non-invasive prenatal testing (NIPT), revealing how the detection of potential genetic anomalies generates significant anxiety. This result aligns with the studies by Dommering et al. [24] and Gregersen et al. [25], who found that the fear of transmitting hereditary diseases, such as retinoblastoma, deeply influences reproductive decisions and the emotional well-being of couples. The uncertainty about the fetus’s health and the possibility of facing difficult decisions during and after pregnancy are common sources of stress.

The emotional impact of specific rare diseases during pregnancy is another highlighted theme. A study by Al-Hasan et al. [11] documents a case of Susac syndrome during pregnancy, highlighting the diagnostic and therapeutic challenges of this rare disease. The need to balance immunosuppression with fetal health creates a high-anxiety environment for the mother. Similarly, de Wolff et al. [21] investigate psychological adaptation in women with peripartum cardiomyopathy, another rare and severe condition. Both studies emphasize the importance of a multidisciplinary approach that includes continuous psychological support to manage the inherent stress and anxiety of these situations.

In the same vein, Beauquier et al. [18] examine the impact of twin-to-twin transfusion syndrome and monochorionicity on prenatal attachment and mothers’ mental health, finding a significant association with post-traumatic stress, anxiety, and depressive symptoms. Blok et al. [20] and Di Mattei et al. [22] examine gestational trophoblastic disease, highlighting the profound emotional impact of this condition. The high levels of anxiety and depression observed underscore the need for continuous psychological follow-up. This approach aligns with the findings of Ireson et al. [26], who advocate for the use of patient-reported outcome measures to effectively address emotional and psychological needs.

Another important aspect is addressing the experiences of women with rare motor disabilities during pregnancy. Dommergues et al. [23] and Smeltzer et al. [32] investigate how these women face unique challenges that affect their emotional well-being and require a specialized care approach. Betegón et al. [19] also demonstrated the effectiveness of a specific program to reduce anxiety in women diagnosed with small-for-gestational-age fetuses, highlighting the importance of tailored psychological interventions.

Finally, attention is drawn to family-centered care and psychological support for parents of children with congenital limb deficiencies (dysmelia). Johnson et al. [28,29] emphasize the need to address feelings of guilt and anxiety during the prenatal and perinatal periods. Overall, the comparison between studies suggests that comprehensive interventions, including psychological support, genetic counseling, and a multidisciplinary approach, are essential to improve the well-being of pregnant women with rare diseases. Specific programs to reduce anxiety, such as those suggested by Betegón et al. [19], and family-centered care, as suggested by Johnson et al. [28,29], are examples of effective strategies that could be more widely adopted.

The importance of the nursing role in addressing this psycho-emotional impact is highlighted. Although silent and indirect, it is simultaneously essential [2]. There are no articles strictly related to nursing and the subject of this review, but most selected articles mention that the nursing role is crucial in these cases [41].

Despite the convergence in identifying high levels of anxiety and depression among pregnant women with rare diseases, the differences in types of diseases and specific needs underscore the importance of a personalized and multidisciplinary approach. Integrating social, psychological, and medical support is essential to address psycho-emotional challenges and improve the quality of life and pregnancy outcomes for this vulnerable group. These studies demonstrate the complexity and need for solutions tailored to the individual circumstances of each patient [4,34].

Reflecting on the limitations of studies addressing psycho-emotional aspects in pregnant women with rare diseases and outlining new lines of research are crucial to advancing this field. One of the main limitations is the scarcity of data due to the rare nature of the diseases studied. This often results in small, less representative samples, which can limit the generalizability of the findings. Additionally, many studies rely on qualitative methods that, while valuable, may not provide the quantitative data needed for robust statistical analyses.

Another significant limitation is the diversity of rare diseases included in these studies. Since rare diseases encompass a wide range of conditions with different etiologies, symptoms, and prognoses, grouping them can dilute the particularities of each condition. This diversity can make general recommendations less precise and fail to adequately address the specific needs of each patient subgroup.

Furthermore, many studies focus on psychological and emotional aspects without fully considering the social, economic, and cultural factors that can influence these women’s experiences. Factors such as access to healthcare, family and community support, and public health policies vary significantly and can impact the psycho-emotional well-being of pregnant women with rare diseases.

Another limitation lies in the lack of longitudinal studies. Most of the available research is cross-sectional, focusing on a specific moment during pregnancy. This limits our understanding of how psycho-emotional issues evolve over time and after childbirth. Longitudinal studies could provide more detailed information about the trajectory of mental health and the long-term impact of interventions.

As for new lines of research, it is essential to develop studies with larger and more representative samples, possibly through international collaborations that can pool data from multiple countries. This would help overcome the sample size limitation and provide more robust and generalizable results.

It is also crucial to include a multidimensional approach that considers not only psychological and emotional factors but also social, economic, and cultural factors. Studies examining how these factors interact and affect the well-being of pregnant women with rare diseases would be valuable for designing more comprehensive and effective interventions.

The use of mixed methodologies combining qualitative and quantitative data could enrich the understanding of these women’s experiences. Longitudinal studies, on the other hand, would provide a more complete view of how psycho-emotional needs change over time and after pregnancy, allowing for more appropriate long-term interventions.

Nursing, as a spearhead, should defend and expand its field of study by presenting independent studies related to the psycho-emotional aspects of pregnant women diagnosed with a rare disease, addressing the approach to these cases and detecting specific needs. At the same time, programs should be developed to coordinate the management of such patients within a multidisciplinary team.

Finally, future research should explore the development and implementation of evidence-based specific interventions. Personalized psychological support programs, the use of digital technologies for continuous monitoring and support, and the training of healthcare professionals to better handle the complexities associated with pregnancy in women with rare diseases are promising areas.

## 5. Conclusions

The reviewed literature demonstrates that pregnant women diagnosed with rare diseases face a wide range of psycho-emotional challenges. The psycho-emotional challenges reviewed include the following: anxiety, stress, depression, and difficulties in prenatal attachment, exacerbated by the uncertainty and complexity of their conditions. It is crucial to provide specialized psychological support and family-centered care to improve their well-being during pregnancy. Undoubtedly, the nursing role must be highlighted as essential in addressing the psycho-emotional realm of these patients, emphasizing competencies such as coordination between healthcare branches, the patient, and their family.

## Figures and Tables

**Table 1:** Search string.

Database	Search String
SCOPUS	((emotional) AND (pregnant AND women)) AND (rare AND disease)
PUBMED	((emotional) AND (pregnant women)) AND (rare disease) (“emoting” [All Fields] OR “emotions” [All Fields] OR “emotions” [MeSH Terms] OR “emotions” [All Fields] OR “emotion” [All Fields] OR “emotional” [All Fields] OR “emotive” [All Fields]) AND (“pregnant women” [MeSH Terms] OR (“pregnant” [All Fields] AND “women” [All Fields]) OR “pregnant women” [All Fields]) AND (“rare diseases” [MeSH Terms] OR (“rare” [All Fields] AND “diseases” [All Fields]) OR “rare diseases” [All Fields] OR (“rare” [All Fields] AND “disease” [All Fields]) OR “rare disease” [All Fields])
SCIELO	((emotional) AND (pregnant women)) AND (rare disease)
CINAHL	((emotional) AND (pregnant women)) AND (rare disease)
COCHRANE LIBRARY	((emotional) AND (pregnant women)) AND (rare disease)

**Table 2:** Methodological evaluation.

Reference	Study Design	Scale	Score
Al-Hasan et al. [11]	Case Study	SCED	9/11
Bakkeren et al. [17]	Observational Study	NEWCASTLE–OTTAWA SCALE	6/8
Beauquier et al. [18]	Observational Study	NEWCASTLE–OTTAWA SCALE	7/8
Betegón et al. [19]	Observational Study	NEWCASTLE–OTTAWA SCALE	5/8
Blok et al. [20]	Observational Study	NEWCASTLE–OTTAWA SCALE	6/8
Wolff et al. [21]	Qualitative Study	CASP	9/10
Di Mattei et al. [22]	Qualitative Study	CASP	8/10
Dommergues et al. [23]	Observational Study	NEWCASTLE–OTTAWA SCALE	8/8
Dommering et al. [24]	Qualitative Study	CASP	10/10
Gregersen et al. [25]	Qualitative Study	CASP	7/10
Hughes et al. [4]	Qualitative Study	CASP	8/10
Ireson et al. [26]	Systematic Review	AMSTAR-2	14/16
Johnson et al. [27]	Systematic Review	AMSTAR-2	13/16
Johnson et al. [28]	Qualitative Study	CASP	9/10
Johnson et al. [29]	Qualitative Study	CASP	8/10
Lekarev et al. [30]	Systematic Review	AMSTAR-2	15/16
Marinello et al. [14]	Qualitative Study	CASP	10/10
Sherrell et al. [31]	Clinical trial	COCHRANE RoB 2	19/23
Smeltzer et al. [32]	Qualitative Study	CASP	7/10
Somanadhan et al. [33]	Qualitative Study	CASP	8/10
Wilson et al. [34]	Case Study	SCED	10/11
Gómez et al. [1]	Systematic Review	AMSTAR-2	10/16
Mills et al. [35]	Qualitative Study	CASP	8/10
Moreau et al. [34]	Systematic Review	AMSTAR-2	14/16
Fiore et al. [36]	Systematic Review	AMSTAR-2	9/16
Di Mattei et al. [37]	Qualitative Study	CASP	9/10
McLaughlin et al. [38]	Systematic Review	AMSTAR-2	12/16
Pascal et al. [39]	Observational Study	NEWCASTLE–OTTAWA SCALE	6/8

**Table 3:** Table of results.

Author	Year	Title	Aim	Result	Conclusion
Gómez et al. [1]	2006	Psychological Disturbances in Women Pregnant	Review the most common psychological alterations in pregnant women	Pregnant women can experience various psychological alterations, including anxiety and depression	Providing psychological support during pregnancy is essential
Dommering et al. [24]	2010	Reproductive decision-making: A qualitative study among couples at increased risk of having a child with retinoblastoma	Investigate reproductive decision-making in couples at increased risk of having a child with retinoblastoma	Couples experience significant ethical and emotional dilemmas	Genetic counseling is crucial to support these couples in decision-making. Emotional aspects such as guilt are altered
Lekarev et al. [30]	2011	Adrenal disease in pregnancy	Review adrenal disease in pregnancy	Adrenal diseases can complicate pregnancy and require specialized management. Highlights the treatment of psychological aspects	A multidisciplinary approach is essential for managing these patients
Di Mattei et al. [22]	2015	Tumori del trofoblasto di origine gestazionale: impatto psicologico e ruolo dei meccanismi di difesa nel percorso di malattia	Examine the psychological impact of gestational trophoblastic tumors in pregnant women and the role of defense mechanisms	Defense mechanisms influence coping and psychological adaptation of patients	Health professionals should consider these mechanisms when treating patients. Emphasizes communication and family support
Johnson et al. [27]	2015	The Impact of Pregnancy on Myotonic Dystrophy: A Registry-Based Study	Evaluate the emotional impact of pregnancy on myotonic dystrophy	Pregnancy can exacerbate symptoms of myotonic dystrophy and affect emotional state	Careful management and monitoring of these patients during pregnancy are necessary
Johnson et al. [28]	2016	Providing family-centred care for rare diseases in maternity services: Parent satisfaction and preferences when dysmelia is identified	Evaluate parent satisfaction and preferences when dysmelia is identified in maternity services	Parents value a family-centered approach and emotional support	Health professionals involved in these cases should understand their needs
Beauquier et al. [18]	2016	Impact of monochorionicity and twin to twin transfusion syndrome on prenatal attachment, post traumatic stress disorder, anxiety and depressive symptoms.	Investigate the impact of monochorionicity and twin-to-twin transfusion syndrome on prenatal attachment and mental health symptoms	Mothers of monochorionic twins exhibit higher levels of stress and anxiety	Providing emotional and psychological support to these mothers is crucial
Smeltzer et al. [32]	2016	Perinatal Experiences of Women With Physical Disabilities and Their Recommendations for Clinicians	Investigate perinatal experiences of women with physical disabilities and their recommendations for clinicians	These women face significant barriers and have recommendations to improve care	Adapting perinatal services to meet these women’s needs is essential
Betegón et al. [19]	2017	A Program Aimed at Reducing Anxiety in Pregnant Women Diagnosed with a Small-for-Gestational-Age Fetus: Evaluative Findings from a Spanish Study	Evaluate a program designed to reduce anxiety in pregnant women with small-for-gestational-age fetuses	The program was effective in reducing anxiety in these women	Psychological interventions can improve mental health in this group of pregnant women
Wolff et al. [21]	2018	Psychological adaptation after peripartum cardiomyopathy: A qualitative study	Explore psychological adaptation after peripartum cardiomyopathy	Women develop varied coping strategies and need emotional support	Psychological and emotional approaches are crucial for managing peripartum cardiomyopathy
Ireson et al. [26]	2018	Systematic review of health-related quality of life and patient-reported outcome measures in gestational trophoblastic disease: a parallel synthesis approach	Review the quality of life and patient-reported outcome measures in gestational trophoblastic disease	The disease significantly impacts the quality of life of patients	Interventions are needed to improve quality of life and psychological support
Johnson et al. [29]	2018	Parent Experiences and Preferences When Dysmelia Is Identified During the Prenatal and Perinatal Periods: A Qualitative Study Into Family Nursing Care for Rare Diseases	Explore parent experiences and preferences when dysmelia is identified during prenatal and perinatal periods	Parents need clear information and emotional support during these periods	Improving communication and support in family nursing care is crucial
Hughes et al. [4]	2019	Psychosocial challenges and needs of women with rare genetic conditions during pregnancy: A qualitative study	Identify the psychosocial challenges and needs of women with rare genetic conditions during pregnancy	These women face unique challenges and require specialized support. Emotional impact detected in stress levels, depression, and psycho-emotional support	Health services must adapt to address these specific needs
Wilson et al. [34]	2019	Sheehan’s syndrome and sickle cell disease: the story of Natasha	Describe a case of Sheehan’s syndrome in a patient with sickle cell disease	Sheehan’s syndrome can significantly complicate the management of sickle cell disease, negatively impacting the emotional state of these pregnant women	A multidisciplinary approach is necessary to manage these complex cases
Moreau et al. [34]	2019	Pregnancy in metabolic diseases with hepatic expression: What risks for the mother and the child?	Evaluate the risks for the mother and child in metabolic diseases with hepatic expression during pregnancy	These diseases can increase the risks of maternal and fetal complications. The emotional impact is evident	Careful management and monitoring of these patients during pregnancy are necessary
Al-Hasan et al. [11]	2020	Susac Syndrome and Pregnancy.	Describe a case of a rare disease during pregnancy	Presentation of a case of a 31-year-old pregnant woman with a new diagnosis of Susac syndrome successfully treated until 36 weeks of gestation with minimal disease burden for both mother and newborn	Highlights the importance of a multidisciplinary approach involving both neurology and maternal–fetal medicine, as well as nursing and health professions addressing emotional impact
Di Mattei et al. [37]	2021	Psychological aspects and fertility issues of GTD	Explore the psychological aspects and fertility issues of gestational trophoblastic disease	Patients experience high levels of anxiety and fertility concerns	Providing psychological support and fertility counseling is crucial
Dommergues et al. [23]	2021	Childbirth and motherhood in women with motor disability due to a rare condition: an exploratory study	Explore childbirth and motherhood experiences in women with motor disabilities due to a rare condition	These women face significant challenges and need specialized support	Providing obstetric care tailored to their needs is essential. Highlights the role of nursing, in this case, midwives
Blok et al. [20]	2022	The psychological impact of gestational trophoblastic disease: a prospective observational multicentre cohort study	Examine the psychological impact of gestational trophoblastic disease	Women with this disease experience high levels of stress and anxiety	Continuous psychological follow-up is necessary for these patients. The role of nursing as a mediator between health branches and the patient is highlighted
Marinello et al. [14]	2022	Exploring patient’s experience and unmet needs on pregnancy and family planning in rare and complex connective tissue diseases: A narrative medicine approach	Explore patient experiences and unmet needs in rare and complex connective tissue diseases during pregnancy and family planning	Patients face multiple challenges and have unmet needs in medical care	Narrative medicine can help better address these patients’ needs
Sherrell et al. [31]	2022	Anxiety, Coping, and Stress: Counseling Parents of Children With a Rare Disease	Provide guidance on counseling parents of children with rare diseases regarding anxiety, coping, and stress	Parents experience high levels of stress and anxiety and need effective coping strategies	Providing psychological support and specialized counseling to these parents is crucial. Nurses know the approach to these interventions
Somanadhan et al. [33]	2022	Living through liminality? Situating the transitional experience of parents of children with mucopolysaccharidoses	Explore the transitional experience of parents of children with mucopolysaccharidoses	Parents live through a liminal experience and face unique challenges	Continuous and specialized support for these parents is needed
Fiore et al. [36]	2022	How I manage pregnancy in women with Glanzmann thrombasthenia	Describe the management of pregnancy in women with Glanzmann thrombasthenia	Careful management and monitoring are crucial to avoid complications	A multidisciplinary approach is necessary to manage these patients
McLaughlin et al. [38]	2022	Interventions for and experiences of shared decision-making underpinning reproductive health, family planning options and pregnancy for women with or at high risk of kidney disease: a systematic review and qualitative framework synthesis	Review the interventions and experiences of shared decision-making in reproductive health for women with or at high risk of kidney disease	Shared decision-making is crucial for these women and improves health outcomes	Improving communication and support in reproductive decision-making is necessary
Mills et al. [35]	2023	Capturing the lived experiences of women with lymphoma in pregnancy: a qualitative study	Explore the experiences of women with lymphoma during pregnancy	Women face significant challenges and need specialized support	Providing obstetric care tailored to the needs of these women is essential
Gregersen et al. [25]	2023	Danish heritable retinoblastoma survivors’ perspectives on reproductive choices: “It’s important for me, not to pass on this condition.”	Explore the perspectives of hereditary retinoblastoma survivors on reproductive choices	Survivors highly value avoiding passing on the disease to their children	Providing genetic and psychological support in reproductive decision-making is crucial
Pascal et al. [39]	2023	Maternal Stress, Anxiety, Well-Being, and Sleep Quality in Pregnant Women throughout Gestation	Evaluate maternal stress, anxiety, well-being, and sleep quality in pregnant women throughout gestation	Stress and anxiety can vary significantly during pregnancy and affect well-being and sleep quality	Providing psychological support and strategies to improve sleep during pregnancy is crucial
Bakkeren et al. [17]	2024	Psychological impact of additional findings detected by genome-wide Non-Invasive Prenatal Testing (NIPT): TRIDENT-2 study	Evaluate the psychological impact of additional findings detected by NIPT in pregnant women	Additional findings can cause stress and anxiety in pregnant women	Post-test counseling should focus on guiding pregnant women during this time of uncertainty

## Data Availability

Data regarding this study is available upon request to the corresponding author.

## Public Involvement Statement

No public involvement in any aspect of this research.

## Guidelines and Standards Statement

This manuscript was drafted against PRISMA MODEL for Systematic Reviews.
